# Cross-Cultural Adaptation of Mediterranean Diet Adherence Screener (MEDAS) Into Moroccan Arabic to Measure the Degree of Mediterranean Diet Adherence

**DOI:** 10.7759/cureus.45556

**Published:** 2023-09-19

**Authors:** Karima Sammoud, Zaynab Mahdi, Kamal Benzaida, Yassine Elrhaffouli, Maryame Yamlahi, Adil Gourinda, Faïza Charif, Fadila Bousgheiri, Hicham Elbouri, Najdi Adil

**Affiliations:** 1 Laboratory of Epidemiology and Public Health, Mohamed VI University Hospital of Tangier, Tangier, MAR; 2 Laboratory of Epidemiology and Public Health, Mohammed VI University Hospital of Tangier, Tangier, MAR; 3 King Fahd School of Translation, University Abdelmalek Essaadi Tangier, Tangier, MAR; 4 Department of Medicine, King Fahd School of Translation, University Abdelmalek Essaadi Tangier, Tangier, MAR; 5 Department of Medicine, Primary Care Facility Level 2 Laoumra, Regional Health Direction of Tangier Tetouan Al Hoceima, Tangier, MAR; 6 Laboratory of Epidemiology and Public Health, University of Medicine and Pharmacy of Tangier, Tangier, MAR; 7 Department of Epidemiology, Faculty of Medicine and Pharmacy of Tangier, Tangier, MAR

**Keywords:** morocco, adherence, the mediterranean diet, cross-cultural validation, medas scale

## Abstract

Background

The Mediterranean diet (MD) has been recognized by several studies as beneficial for health improvement. The degree of adherence to this diet has also been evaluated using several scales, particularly time-consuming measures such as the Food Frequency Questionnaire (FFQ). This study aims to (a) adapt into Moroccan Arabic the 14-item Mediterranean Diet Adherence Screener (MEDAS), which is a simple and brief tool that assesses the degree of diet adherence and was used in the Prevencion con Dieta Mediterranea (PREDIMED) study, and (b) determine its psychometric properties.

Methods

MEDAS consists of 12 questions on food frequency and two on dietary habits, with each question scoring 0 or 1. To translate and adapt the scale, Beaton et al.’s six-step cross-cultural adaptation process guidelines were followed.

The screener's psychometric properties were tested on staff at the CHU Mohammed VI (Tangier), i.e., the hospital's administrative and maintenance staff, excluding medical and paramedical personnel. Internal consistency was evaluated using the Kuder-Richardson 21 (K-R 21) formula, while test-retest reliability was assessed using the intraclass correlation coefficient (ICC). Moreover, criterion validity was performed using the Spearman correlation between the MEDAS and the MedQ-Sus scores. Discrimination performance was also tested using the receiving operating characteristic (ROC) curve.

Results

The validation study included 160 participants who completed both questionnaires. The K-R 21 formula estimated strong internal consistency in the range of 0.851. The ICC of test-retest reliability was significant at 0.876 95% CI [0.831-0.909]. The MEDAS score correlated significantly with the comparative MedQ-Sus score (Spearman's rho = 0.494 95% CI [0.363-0.606], p < 0.001). Also, MEDAS can strongly distinguish between MD adherence and non-adherence (optimal cut-off = 7.5, sensitivity 0.81, specificity = 0.57), with an area under the curve (AUC) value of 0.743 95% CI [0.667-0.819], p < 0.001.

Conclusion

The results showed that MEDAS is a valid and time-saving instrument for assessing adherence to the MD in the Moroccan population.

## Introduction

The Mediterranean diet (MD) is considered beneficial for health improvement by several studies [1-9]. The degree of adherence to this diet has been evaluated using numerous measures, particularly time-consuming scales such as the Food Frequency Questionnaire (FFQ), which includes several items, more than 100 in most cases, as well as 24-hour recalls [10,11]. Since there is no simple and short-form tool for assessing adherence to the MD in Morocco, the possibility of a valid and reliable Moroccan version of such an instrument would be useful. For this purpose, the validated 14-item Mediterranean Diet Adherence Screener (MEDAS) was chosen to assess the adherence of the northern Moroccan population [12,13]. Adapting this particular instrument for cultural purposes rather than creating a new one has several advantages, including reduced time and cost. MEDAS has also been used in many studies and translated into different languages [10,13-15]. Thus, this study’s objective is to adapt this scale to Moroccan Arabic and determine its psychometric properties.

## Materials and methods

Participants

In February 2023, the Mohammed VI Hospital (Tangier) staff were invited to participate in the validation study, i.e., hospital administrative, maintenance, cleaning, and porter staff. Medical and paramedical staff were excluded. The sample size was calculated at 140 based on a subject/question ratio of 10:1 [16], using stratified random sampling based on non-medical hospital departments [16]. The participants were required to answer the translated questionnaire twice; one week elapsed between their first and second responses. Moreover, the MedQ-Sus questionnaire was used as a reference instrument.

The scale was sealed with a self-selected code to ensure confidentiality. The codes were in the form of two stickers, and each participant had two stickers with the same code. To apply the test-retest strategy, the respondents used the first sticker on the envelope provided during their initial participation and the second sticker during their subsequent participation.

Eligible participants in the study had to be over 18 years of age, on a regular diet, not taking any medication, and with no health problems affecting their diet. They were informed of their role in the study and formally consented before being interviewed.

Translation and cultural adaptation of the MEDAS score

MEDAS is a 14-question scale that was first used in the Prevencion con Dieta Mediterranea (PREDIMED) trial, a randomized primary cardiovascular prevention study conducted at 11 Spanish enrollment centers [12,13,17]. Beaton et al.’s six stages of cross-cultural adaptation guided the translation process [18]. The first stage involved translating the English version into Moroccan Arabic, which is widely spoken by Moroccans. This process was carried out separately by two bilingual translators, with both versions in Moroccan Arabic; then, a synthesis of the two versions followed. A back-translation was also accomplished by two other translators, who were unfamiliar with the scale. Two back-translation versions were obtained, both of which were reviewed by a multidisciplinary expert committee composed of methodologists, language specialists, and translators. The final version was produced in Moroccan Arabic and English; it was tested on 11 interview subjects to ensure a thorough understanding of the items.

Mediterranean diet adherence screener (MEDAS)

MEDAS is composed of 14 items that are designed to measure the degree of adherence to the MD. It is an extended version of a nine-item scale [19]; the additional five items are concerned with (i) the use of olive oil in food preparation; (ii) preference for white or red meats; (iii, iv) frequency of nuts and sweet drink consumption; and (v) frequency with which vegetables or pasta are prepared with tomato sauce, onions or garlic, and leeks processed in olive oil.

The instrument includes 12 questions on food frequency and two on dietary habits, with each question scoring 0 or 1 [13]. The total MD score ranges from 0 (no adherence) to 14 (high adherence) [12].

MedQ-Sus

MedQ-Sus is a questionnaire developed by Ruggeri et al. [20]. It is a short, validated instrument that measures adherence to the MD, excluding alcohol intake, and is designed for all adult population groups. MedQ-Sus consists of eight questions on the consumption of the following eight food groups: cereals and cereal products, legumes, fresh vegetables, fresh fruit, dairy products, fish and fish products, meat and meat products, and olive oil. Each food group is assigned a quantitative score (from 0 to 2), and the total MedQ-Sus score ranges from 0 (no adherence) to 16 (strong adherence). It is simple to use and does not include any lengthy questions, only the food category name and the recommended serving size per day or week. Hence, it is appealing for use as a reference tool without having to adapt it semantically and culturally. The MedQ-Sus was used in a large population, the majority of whom found it difficult to estimate their food intake in grams; thus, units of measurement such as bowls, dishes, and spoons were employed instead of portion sizes in grams.

Statistical analysis and psychometric properties

All analyses were performed using IBM SPSS version 25 (IBM Corp., Armonk, NY). Internal consistency was tested using the Kuder-Richardson 21 (K-R 21) formula [21-23], and test-retest reliability was assessed using the intraclass correlation coefficient (ICC). Criterion validity was performed using the Spearman correlation between the MEDAS and MedQ-Sus scores. Discrimination performance was evaluated via the receiving operating characteristic (ROC) curve. To perform the ROC analysis, the score was coded into two categories according to its median-related position; data was coded 0 if it was lower or higher than the median.

Ethical considerations

The approval of the Ethics Committee of the University Hospital of Tangier was obtained on February 2, 2023, under the number 17/2023. Before the subjects were included, the study’s aim was clarified to all participants, and their informed consent was obtained.

## Results

Translation process

The translation and adaptation process went smoothly, except for some red meat-related items, namely item 5. The translators agreed that the term “hamburger” is the most appropriate to be translated as “kafta” (ground meat), given that some people may not understand that hamburger is simply ground meat prepared differently. Moreover, given the Moroccan context, the term “ham” or smoked pork meat was translated as “kasher” since it is the equivalent product widely consumed by the Moroccan population. Also, it was difficult to translate the term “sofrito” for item 14, as Moroccans have no equivalent name for this sauce, even though they use the same ingredients during meal preparations. Therefore, sofrito was kept only to avoid deviating from the scale’s original version.

Participant characteristics

This study included 160 subjects, with a mean age of 28.59 years (SD = 7) and a female predominance of 66.9% (sex ratio F/M = 2.01). Although most of the participants were single (70%), they mostly lived with their families (60%). Also, most respondents in the sample did not smoke (80.5%) and were of normal weight (65.4%) (Table 1).

**Table 1:** Participants' characteristics

Variables	Values (n=160)
Age (Median, range)	27(19-59) years
Sex	
Male	33.1%(53)
Female	66.9%(107)
Marital status	
Single	70.6%(113)
Married	25%(40)
Divorced	4.4%(7)
Cohabitation status	
Living alone	40%(64)
Living in a family	60%(96)
Education level	
Unschooling	1.3%(2)
Elementary school	1.9%(3)
High school	11.3%(18)
Academic level	85.6%(137)
Smoking status	
Habitual smoker	13.8%(22)
Non-smoker	80.5%(128)
Passive smoker	3.8%(6)
Weaned smoker	1.9%(3)
Alcohol intake	
Yes	3.1%(5)
No	96.9%(155)
Body mass index	
Underweight	5.7%(9)
Normal weight	65.4%(104)
Overweight	23.3%(37)
Obesity	5.7%(9)

Acceptability

The questionnaire was generally acceptable, and the average time required to complete it was brief. In addition, there were no missing responses.

Reliability

Internal Consistency

Since MEDAS is composed of items with dichotomous scores of 0 or 1, the homogeneity of the items was tested using the Kuder-Richardson 21 formula. A strong internal consistency at 0.851 was determined.

Reproducibility

The Moroccan version’s test-retest reliability was assessed using the intraclass correlation between the two screener administrations’ total scores. A significant ICC for test-retest reliability at 0.876, 95% CI [0.831-0.909] was attained.

Criterion validity

The MedQ-sus questionnaire was employed for the validation study. The respondents completed both screeners. A high Spearman correlation coefficient (rho = 0.494, 95% CI [0.363-0.606], p < 0.001) was found between the MedQ-Sus and MEDAS scores.

Discriminatory performance

**Figure 1:**
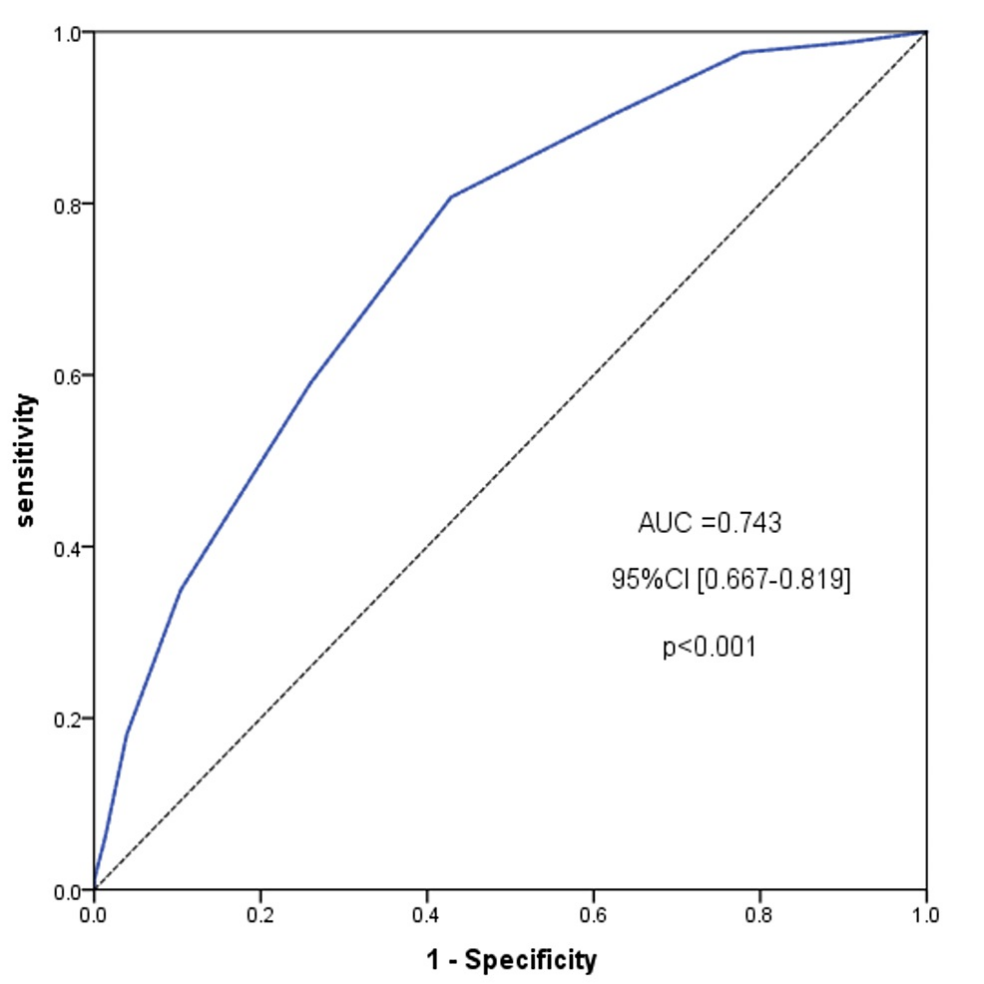
Receiving operating characteristic (ROC) curve for the MEDAS scale

ROC analysis revealed that MEDAS was good in discerning MD-adherent and non-adherent subjects. With an area under the curve (AUC) value of 0.743, 95% CI [0.667-0.819], p < 0.001 (Figure 1), the AUC is a useful parameter for summarizing the ROC curve. Using the point at the corner closest to 0.1 in the ROC curve, the optimal threshold calculated was 7.5, which corresponded to a sensitivity of 0.81 and a specificity of 0.57. Taking this threshold into account, the number of subjects who followed the MD was 100 (62.5%), whereas those who did not adhere were 60 (37.5%).

## Discussion

The MD is prevalent in many Mediterranean-coastal countries. Several studies have been conducted to evaluate MD adherence in both Mediterranean and non-Mediterranean regions, where the diet has been found appealing for its role in preventing cardiovascular, metabolic, and degenerative diseases [6,24,25].

Several scales, both long and short, have been developed to assess MD adherence. In this study, the researchers chose an existing instrument rather than develop a new one to avoid wasting time and resources. The selected tool has been widely used in several studies to measure MD adherence and was first employed in the PREDIMED randomized trial by Miguel A. Martinez-Gonzalez [26].

The translation process was challenging due to the diverse terms found in different region-based Moroccan dialects. The translators agreed to choose terms that were suitable across all Moroccan regions and on how foods not consumed by Moroccans, such as pork, should be adapted. All the changes were approved by an expert committee, and the final version was tested on 11 people, all of whom found the questionnaire to be well-translated and understandable. 

As an agricultural country and having two coastlines: the Mediterranean and the Atlantic, the main components of the MD are common in Morocco. Thus, the respondents are familiar with the screener’s proposed foods, making the survey easy and quick to conduct.

In comparison to other studies, the scale has credible psychometric properties; it has high internal consistency, with a K-R 21 coefficient of 0.851, as well as high reliability based on the test-retest strategy [14,27]. The time interval between the two screener administrations was one week; this is acceptable, as it is neither too short to avoid recall bias, nor too long to avoid switching to a different diet or other variations [28].

The instrument’s validity has been verified using a validated reference questionnaire, and the correlation between the two scale scores was significant. The ROC analysis also revealed that MEDAS has a good discriminatory power and a calculated optimal cut-off of 7.5, with a corresponding sensitivity of 0.81 and a specificity of 0.57. These results are comparable to those obtained in previous studies [12,13].

One of this study’s strengths is the adoption of a short, easy-to-use instrument that is apt for epidemiological studies and can be applied in clinical practice. Another of its strong points is the lack of similarity between MEDAS and MedQ-Sus, preventing overestimation of the screener’s validity. MEDAS can also be used to help individuals identify variations between different food groups to promote MD adherence. In addition, the use of a standardized scale would enable future researchers to compare results globally and conduct meta-analyses.

This study, however, has certain limitations. First, the two instruments used to assess dietary intake gathered self-reported data, which may be imprecise. Second, the sample was composed of highly educated individuals, which does not represent the majority of Morocco, where illiteracy is common.

## Conclusions

In this study, the validated Moroccan Arabic MEDAS is a good and quick-to-implement screener that reliably measures the degree of MD adherence compared with long-form ones. With its valid psychometric properties and well-translated terms equivalent to the Moroccan population’s main food intake, this version can be used to assess the level of MD adherence in Morocco, both for research and clinical management.

## Appendices

**Table 2:** MedQ-Sus questionnaire

Cereals & cereal products (including whole, sweets excluded) 130 g	<1 portion/day	1–1.5 portion/day	>1.5 portion/day
Legumes 70 g	<1 portion/week	1–2 portion/week	>2 portion/week
Fresh vegetables 100 g	<1 portion/day	1–2.5 portion/day	>2.5 portion/day
Fresh fruit 150 g	<1 portion/day	1–2 portion/day	>2 portion/day
Dairy products 180 g	<1 portion/day	1–1.5 portion/day	>1.5 portion/day
Fish & fish products (except shellfish and crustaceans) 100 g	<1 portion/week	1–2.5 portion/week	>2.5 portion/week
Meat & meat products 80 g	<1 portion/day	1–1.5 portion/day	>1.5 portion/day
Olive oil	Occasional Consumption (<4 spoons/day)	Regular Consumption (about 4–5 spoons/day)	Frequent Consumption (>4 spoons/day)
